# Facile Fabrication of Wood-Derived Porous Fe_3_C/Nitrogen-Doped Carbon Membrane for Colorimetric Sensing of Ascorbic Acid

**DOI:** 10.3390/nano13202786

**Published:** 2023-10-18

**Authors:** Sadaf Saeedi Garakani, Miao Zhang, Dongjiu Xie, Anirban Sikdar, Kanglei Pang, Jiayin Yuan

**Affiliations:** 1 Department of Materials and Environmental Chemistry, Stockholm University, 10691 Stockholm, Sweden; sadaf.saeedigarakani@mmk.su.se (S.S.G.); miao.zhang@mmk.su.se (M.Z.); anirban.sikdar@mmk.su.se (A.S.); kanglei.pang@mmk.su.se (K.P.); 2 Department for Electrochemical Energy Storage, Helmholtz-Zentrum Berlin für Materialien und Energie, Hahn-Meitner Platz 1, 14109 Berlin, Germany; dongjiu.xie@helmholtz-berlin.de

**Keywords:** iron carbide nanoparticles, nitrogen-doped carbon, wood-derived carbon, colorimetric detection, ascorbic acid

## Abstract

Fe_3_C nanoparticles hold promise as catalysts and nanozymes, but their low activity and complex preparation have hindered their use. Herein, this study presents a synthetic alternative toward efficient, durable, and recyclable, Fe_3_C-nanoparticle-encapsulated nitrogen-doped hierarchically porous carbon membranes (Fe_3_C/N–C). By employing a simple one-step synthetic method, we utilized wood as a renewable and environmentally friendly carbon precursor, coupled with poly(ionic liquids) as a nitrogen and iron source. This innovative strategy offers sustainable, high-performance catalysts with improved stability and reusability. The Fe_3_C/N–C exhibits an outstanding peroxidase-like catalytic activity toward the oxidation of 3,3′,5,5′-tetramethylbenzidine in the presence of hydrogen peroxide, which stems from well-dispersed, small Fe_3_C nanoparticles jointly with the structurally unique micro-/macroporous N–C membrane. Owing to the remarkable catalytic activity for mimicking peroxidase, an efficient and sensitive colorimetric method for detecting ascorbic acid over a broad concentration range with a low limit of detection (~2.64 µM), as well as superior selectivity, and anti-interference capability has been developed. This study offers a widely adaptable and sustainable way to synthesize an Fe_3_C/N–C membrane as an easy-to-handle, convenient, and recoverable biomimetic enzyme with excellent catalytic performance, providing a convenient and sensitive colorimetric technique for potential applications in medicine, biosensing, and environmental fields.

## 1. Introduction

Ascorbic acid (AA), also called vitamin C, is a crucial water-soluble vitamin. Its antioxidant properties are effective for developing the immune system, preventing and treating some diseases such as cancer, and creating collagen in various tissues in the human body [1,2]. Since the human body cannot generate this vital vitamin, gaining enough AA from the daily diet and health supplements is essential. Consequently, developing quick and straightforward approaches to determine the concentration of AA with high selectivity and accuracy is required in different fields, e.g., pharmacy and food industries [3]. So far, several methods, for instance, high-performance liquid chromatography (HPLC) [4], capillary electrophoresis [5], fluorescence [6], and electrochemistry [7], have been used to evaluate the AA concentration. Compared with other techniques, the colorimetric assay has recently gained widespread attention as a reliable biosensor due to its distinctive advantages such as rapid detection, simplicity, superb sensitivity, low cost, and facile optical detection with the naked eye [8]. To develop a quick colorimetric sensor, peroxidase, a kind of natural enzyme, plays a pivotal role thanks to its high catalytic efficiency and specificity [9]. However, high cost, low stability, brief shelf life, and harsh environmental conditions significantly hamper its practical use [10]. To overcome these challenges, researchers have developed nanomaterial-based artificial enzymes (“nanozymes”) that can mimic the functions of natural enzymes [11]. So far, a collection of nanomaterials, e.g., metallic nanoparticles [12,13], carbon-based nanomaterials [14,15], and metal–organic frameworks (MOFs) [16], have been successfully crafted, which demonstrated the inherent peroxidase-like function.

Recently, Fe_3_C, a member of the iron carbide family, indicated outstanding peroxidase-like catalytic activity because of its high redox capacity, a large number of active sites, and superior surface reactivity [17,18]. Especially, the ultrafine Fe_3_C-based nanoparticles possess highly exposing active sites and could therefore be considered as a promising catalytic agent. Nevertheless, the strong aggregation tendency of Fe_3_C nanoparticles often leads to loss of surface area, reducing their catalytic capacity [19,20]. Dispersing Fe_3_C nanoparticles over a porous conducting matrix, especially heteroatom-doped porous conductive carbon, could be an effective strategy to minimize nanoparticle aggregation. In this composite catalyst, the carbon matrix plays a dual role: first, it maintains the high catalytically active surface area for Fe_3_C; second, it endows the catalyst with sufficient conductivity and chemical stability due to the carbon component [21,22]. In addition, the heteroatom doping of carbon may synergistically boost the catalytic process through tuning the local electronic and chemical structure [23]. Therefore, it is of high interest to explore synthetic strategies to synthesize ultrafine Fe_3_C nanoparticles dispersed over a heteroatom-doped porous carbon matrix that can exhibit peroxidase-like activity.

Herein, we established a facile wood-based method for synthesizing a nitrogen-doped porous carbon membrane containing well-dispersed Fe_3_C nanoparticles (termed “Fe_3_C/N–C”) through sequential pyrolytic treatments. The as-prepared Fe_3_C/N–C catalyst illustrated an excellent characteristic peroxidase-like activity to 3,3′,5,5′-tetramethylbenzidine (TMB) oxidation due to the ultrafine Fe_3_C nanoparticles and their corresponding cooperative interfacial effect between Fe_3_C nanoparticles and the nitrogen-doped carbon matrix. Because of the elevated catalytic activity, a convenient colorimetric assay of AA has been developed. It has been proven that the optimized Fe_3_C/N–C composite catalysts are impressive peroxidase mimics, which exhibit promising opportunities in the fields of biosensors and biomedicine.

## 2. Experimental Section

**Materials.** Balsa wood was purchased from Material AB, Sweden. 1-Vinylimidazole (99%) and sodium tetrafluoroborate were obtained from Alfa Aesar, Karlsruhe, Germany. Potassium hexafluorophosphate was purchased from Acros Organics, France. Bromoacetonitrile (95%) was purchased from TCI, Europe. Lithium bis(trifluoromethane sulfonyl)imide (LiTFSI, 99.95%) was purchased from Io-li-tec, Heilbronn, Germany. Sodium chlorite, sodium acetate, 3,3′,5,5′-tetramethyl-benzidine (TMB), L-ascorbic acid, and iron (III) chloride were purchased from Sigma-Aldrich, Darmstadt, Germany. N,N-dimethylformamide (DMF) was obtained from Honeywell, Germany. H_2_O_2_ was purchased from VWR, Leuven, Belgium. All chemicals were used without any further purification. Solvents were of analytical grade.

**Poly(ionic liquid) (PIL) synthesis.** The polymer precursor with Br^−^ as counteranion, poly(1-cyanomethyl-3-vinylimidazoulim bromide) (PCMVImBr), was synthesized in reference to our earlier published procedure [24]. Its chemical structure was characterized through proton nuclear magnetic resonance (^1^H-NMR) spectroscopy, as demonstrated in Figure S1. Poly(1-cyanomethyl-3-vinylimidazoluim bis(trifluoromethane sulfonyl)imide) (PCMVImTFSI) was made through an anion metathesis reaction between PCMVImBr and LiTFSI in their aqueous solution. In a standard anion-exchange procedure, an aqueous solution of LiTFSI was added dropwise to an aqueous solution of PCMVImBr at a concentration of 1 wt%. The final TFSI/Br molar ratio was set as 1.15/1. The precipitate was filtered off and washed with pure water several times, and finally dried at 70 °C under vacuum until constant weight.

**Wood delignification.** Balsa wood with a density of 123 kg m^−3^ was sliced into thin membranes with controllable thickness using cutting equipment (secotom-50). The cutting direction was maintained perpendicular to the growth direction of the trunk. Before chemical treatment, all wood slices were dried at 80 °C in an oven for 10 h. To extract hemicellulose and lignin, a well-established method was employed using sodium chlorite (1 wt%) with acetate buffer solution (pH 4.6) for 6 h at 80 °C. After the treatment, the membranes were rinsed with deionized water and ethanol and dried at room temperature until constant weight.

**Preparation of the Fe_3_C/N–C catalyst.** Amounts of 0.200 g of the as-synthesized PCMVImTFSI and 0.030 g of iron (III) chloride were thoroughly dissolved in 2 mL of DMF until a homogenous solution was achieved. The 0.600 g delignified wood membranes were coated with the solution and dried at 80 °C for 2 h. The obtained membranes were immersed in a 0.25 wt% aqueous ammonia solution for 2 h to generate a porous polymeric structure on the cells of the wood. Then, the obtained membranes were washed with pure water several times. It was finally dried under ambient conditions until a constant weight. Next, the prepared membranes were carbonized as the following procedure in a tube furnace. First, the membranes were heated at 300 °C in nitrogen flow for 1 h, heated to 600 °C, and maintained at this temperature for 1 h; finally, they were heated to 900 °C and kept for 10 min. For comparison, the membrane was also carbonized at different final temperatures, including 700, 800, and 1000 °C. Finally, the Fe_3_C/N–C catalysts were obtained.

**Peroxide catalytic activity.** Typically, 20 µL of the as-prepared Fe_3_C/N–C suspension (3 mg mL^−1^) and 20 µL of TMB solution (15 mM) in DMSO were added into 3 mL of acetate buffer solution (pH 4) containing H_2_O_2_ (50 mM) at room temperature. The oxidation of TMB by the catalyst was studied by monitoring the absorbance peak at 652 nm after 10 min of reaction. The other groups, including TMB + H_2_O_2_ and TMB + catalyst (Fe_3_C/N–C) at the same concertation as mentioned, were selected as the controls. Photographs of the reaction solution were obtained, and UV-Vis absorbance was collected using a UV-Vis-NIR spectrophotometer (Agilent Technologies, Santa Clara, CA, USA). Under the above-mentioned concentrations, the catalyst’s pH tolerance was investigated in a wide range (2.0~11.0) at room temperature. Similarly, temperature tolerance was examined in the reaction by altering the temperature between 25 and 60 °C in acetate buffer (pH 4).

**Kinetic analysis.** The kinetics of the reaction was considered by monitoring the absorbance variation at 652 nm at one-minute intervals in a scanning mode. The steady-state kinetics was analyzed using H_2_O_2_ and TMB as substrates. The tests were performed by altering the TMB concentration at a fixed H_2_O_2_ concentration or contrariwise. The Michaelis–Menten equation was applied to determine the kinetic parameters (Equation (1)).

(1) $$\mathrm{Michaelis}–\mathrm{Menten}\;v=V_{max}\times \left[S\right]/(K_{m}+\left[S\right])$$

where *v*, *V_max_*, [*S*], and *K_m_* represent the initial velocity, maximum reaction velocity, substrate concentration, and Michaelis constant, respectively. The molar attenuation coefficient of TMB at 652 nm was 39,000 M^−1^cm^−1^. The tests were carried out in a colorimetric dish of 1 cm in thickness.

**Detection of ascorbic acid.** AA detection was studied as follows: 15 µL of H_2_O_2_ sample, 60 µL of TMB solution, and 20 µL of catalyst dispersion in acetate buffer (pH 4.0) with a final volume 500 µL reacted for 15 min at room temperature, and the blue color of oxTMB was observed. Then, the ascorbic acid samples at different concentrations of 0–200 µM were individually added to the blue solution, and the UV-Vis absorbance at 652 nm was monitored. Similarly, the experiment was repeated by replacing AA with various reagents such as sulfite, citric acid, glycine, serine, alanine, aspartic acid, tartaric acid, arginine, and saccharides like glucose, lactose, and fructose.

**Characterization.** Phase structure was examined on an X-ray diffractometer PANalytical X’Pert Pro (Malvern Instruments, Malvern, UK) applying Cu K_α_ radiation (λ = 1.5418 Å) between 5° and 80° at a scan rate of 0.2°/min. Chemical bonding characterizations were monitored through ESCALAB 250Xi X-ray photoelectron spectroscopy (XPS) (USA). The morphology of samples was investigated using a JEOL 7000F (JEOL Ltd., Tokyo, Japan) scanning electron microscope (SEM) operated at 10 kV. Samples were sputtered by a thin gold layer for 60 s before the examination. Energy-dispersive X-ray (EDX) mapping was taken on the SEM using an EDX spectrometer. The particle size was determined through transmission electron microscopy (TEM) using a JEOL JEM-2100 (JEOL GmbH, Eching, Germany) operated at 200 kV. The nitrogen sorption isotherms at 77 K were performed using the Micromeritics ASAP 2020 (Accelerated Surface Area and Porosimetry system, Germany). Before the sorption experiments, all samples were degassed for 7 h at 373 K under vacuum. The Brunauer–Emmett–Teller (BET) equation was used to calculate the specific surface area. Thermogravimetric analysis (TGA) experiments were carried out at a heating rate of 10 °C min^−1^ from 50 °C to 900 °C under air flow using a TA Instruments Discovery TG. Raman spectroscopy was performed on a Horiba Labram HR system with 532 nm laser excitation. ^1^H-NMR spectra were performed at room temperature using a Bruker DPX-400 spectrometer operating at 400 MHz. DMSO-*d*_6_ was employed as an NMR solvent for the measurement. Additionally, the catalytic attributes were examined through ultraviolet-visible (UV-Vis, Agilent Technologies UV-Vis-NIR spectrophotometer, Petaling Jaya, Malaysia) measurement.

## 3. Results and Discussion

Fe_3_C nanoparticle-functionalized nitrogen-doped porous carbon membranes, termed Fe_3_C/N–C, were synthesized via a facile pyrolysis of Balsa wood in the presence of a mixture of poly(ionic liquid) and FeCl_3_ as N/Fe sources. It is known that the chemical nature and microstructure of precursors strongly affect the physiochemical properties and chemical composition of heteroatom-doped carbon products [25]. Thus, wood carrying the elegant form of interconnected and oriented channel-like pores, in addition to its renewability and low cost, stands out as an attractive source for conductive porous carbon matrix [26,27]. Obviously, various types of wood possess different internal porous structures, and Balsa wood was chosen here as a carbon precursor owing to its multi-channel hierarchically porous structure. Furthermore, due to its high nitrogen content, poly(ionic liquid) (PIL) as the polymerization product of ionic liquids can serve as the source of nitrogen that endows porous carbons with target N doping; in addition, the PIL can induce the formation of extra porous carbon structures through its catalytic degradation of biomass [28]. In comparison to other types of polymers, PILs can be more thermally stable to secure a high carbonization yield [29,30], can be rich in heteroatoms of different types that can dope carbon products [31,32], and can molecularly disperse the iron precursor to secure uniform formation of Fe_3_C nanoparticles in the porous carbon matrix [33]. To note, the cation–anion pair in PIL is one of the key parameters in creating the tiny pores to accommodate active sites for catalysis [24]. Using the wood slice and PIL as precursors readily enables the formation of a thin porous carbon membrane, which is effective for the enrichment of AA and proper for practical use in in situ detection. More significantly, as a heterogeneous catalyst at a macroscopic size, the composite membrane can be recycled at will from the liquid reaction system [34,35].

In a typical synthetic run, after chemical treatment of Balsa wood to remove lignin and hemicellulose, a mixture of an aqueous solution of FeCl_3_ and PCMVImTFSI (TFSI denotes the counteranion) was coated onto the wood cells’ surface through straightforward wet-impregnation and drying under ambient conditions to constant weight. PILs are well known for their high surface activities and attach themselves efficiently onto the wood surface via multiple intermolecular interactions, e.g., hydrogen bonding and van der Waals interaction. Because of the ionic complexation of the iron cation with the -CN unit and the ion pair in the PIL, the FeCl_3_ can be embedded in PIL and uniformly anchor as a coating layer onto the porous wood surface [36]. This step is critical for forming the iron carbide nanoparticles and minimizing their severe aggregation caused by the uneven distribution of iron precursors. Afterward, a pyrolysis treatment at various carbonization temperatures under nitrogen was performed to generate the target Fe_3_C/N–C membranes.

**Figure 1:**
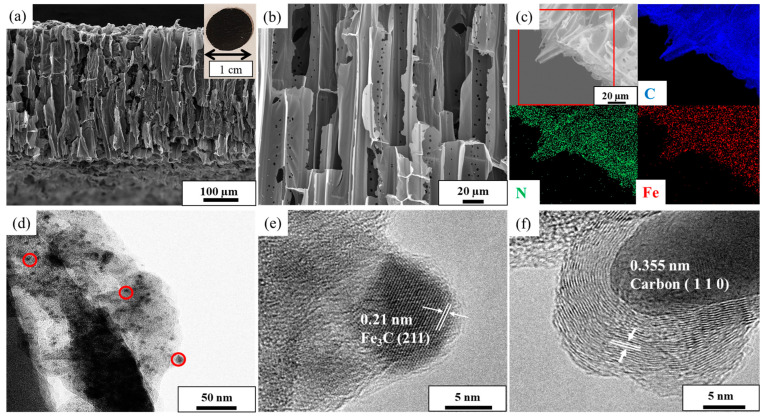
(**a**,**b**) Cross-sectional SEM images of Fe_3_C/N–C, respectively. The inset in (**a**) shows the photograph of the final membrane prepared at 900 °C. (**c**) Elemental mapping of different elements in Fe_3_C/N–C, (**d**) TEM image of Fe_3_C/N–C, and (**e**,**f**) HR-TEM images of Fe_3_C/N–C.

Because of the benefit of the facile scalable synthetic method, the technique can properly tailor the hierarchical membrane with a controllable composition and microstructure. Since the enzyme-mimicking activities of carbon materials are obviously affected by the final carbonization temperatures, the alteration in catalytic activity against the carbonization temperature was studied first between 700 °C and 1000 °C, wherein 900 °C was identified as the optimum temperature; thus, studies thereafter were based on this sample (Figure S2). As shown in the inset of Figure 1a, a photograph of a spherical carbon membrane of Fe_3_C/N–C of 1 cm in diameter is presented. Since the Fe_3_C/N–C membrane is cuttable by a normal cutter, it can be easily produced in different sizes and shapes. Next, the morphology of the as-synthesized catalyst was studied using scanning electron microscopy (SEM) and transmission electron microscopy (TEM). The cross-sectional SEM images of Fe_3_C/N–C (Figure 1a,b and Figure S3) show that the 3D porous structure of the Balsa wood with well-aligned microchannels are completely preserved after the pyrolysis process [37]. This open channel in the 3D porous carbon framework with low tortuosity can significantly lower the diffusion length, resulting in smooth and fast mass transportation to and from the active sites [38]. Besides the large-sized channels, the SEM images further reveal the presence of macropores of 1.75 ± 0.5 µm sitting directly on the cell wall of the carbon, which interconnect parallel channels of the carbon framework.

The decoration of Fe_3_C nanoparticles over the porous carbon framework was revealed by TEM images. Figure 1d displays nanoparticles of Fe_3_C homogenously dispersed over the thin carbon nanosheets without agglomeration. Importantly, the histogram of the nanoparticles’ size distribution (Figure S4) shows that the average diameter of the Fe_3_C nanoparticles is small, 4.9 ± 2.5 nm. The ultra-small nanoparticles embedded in the porous carbons possess rich accessible active sites and therefore are beneficial to their catalytic performance [39]. To study the role of PIL on the formation of dispersed Fe_3_C nanoparticles, a control sample was synthesized without PIL under the same condition. The TEM image of the control sample demonstrates the aggregation of nanoparticles into a large size of 39 ± 19 nm (Figures S5 and S6), confirming the cooperative role of PIL in the formation of well-dispersed Fe_3_C nanoparticles. To further analyze the successful formation of the Fe_3_C/N–C catalyst, we switched to energy-dispersive X-ray (EDX) mapping and high-resolution TEM (HR-TEM) images. First, the EDX mapping shows the microscopic uniform distribution of Fe, N, and C elements across the catalyst surface (Figure 1c), in accordance with the observed homogenous distribution of Fe_3_C nanoparticles within the porous carbons through TEM. The presence of the N element supports the successful doping of the carbon framework with nitrogen atoms owing to the decomposition and doping of the carbon matrix by PIL at high temperature (>500 °C) [40]. From the HR-TEM images of Fe_3_C/N–C (Figure 1e,f), we can clearly observe two distinct crystalline phases with lattice spacings of 0.21 nm and 0.36 nm corresponding to the crystallographic planes (220) of Fe_3_C and (002) of graphitic carbon, respectively [41,42]. The presence of a graphitic phase (002) with an interlayer spacing of 0.36 nm suggests the occurrence of a graphitization process of the wood/PIL mixture at a temperature of 900 °C due to the catalytic role of PIL to biomass during pyrolysis [28].

**Figure 2:**
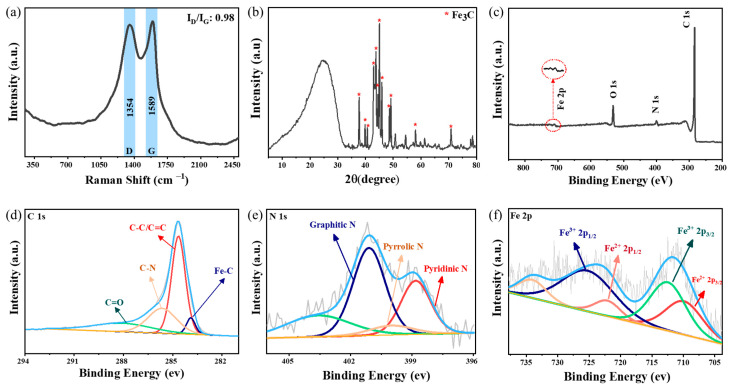
(**a**) Raman spectrum, (**b**) XRD pattern, and (**c**) XPS full survey spectrum of the obtained Fe_3_C/N–C; (**d**–**f**) XPS survey spectra of C 1s, N 1s, and Fe 2p, respectively.

The degree of graphitization and the phase structure information of the carbons in Fe_3_C/N–C were studied using Raman spectroscopy. Figure 2a shows the appearance of two distinct bands at 1350 cm^−1^ and at 1590 cm^−1^, which are assigned, respectively, to the D-band originating from the disorder in carbon atoms and structural defects, and the G-band, which is attributed to the ordered carbon structures [31,43]. The intensity ratio of the D band to the G band was quantitatively calculated to be 0.98. Importantly, graphitization improves the transport of electrons through the carbonized porous wood, which for this sample is measured to be 232 ± 16 S/m as the apparent conductivity (due to the presence of pores). As a result, this graphitic wood could serve as a conductive matrix to favor peroxidase catalytic activity.

The phase structure of as-synthesized Fe_3_C/N–C catalysts was analyzed via an X-ray diffraction (XRD) study. In Figure 2b, the diffraction peaks at 25.0°, 42.9°, 44.4°, 54.4°, and 77.9° could be assigned to the diffraction planes of (002), (100), (101), (004), and (110) of the graphitic phase (JCPDS: 41-1487), respectively. The diffraction peaks at 37.7°, 39.8°, 40.6°, 42.8°, 43.8°, 44.5°, 45.0°, 45.9°, 48.6°, and 49.1° can be attributed to the crystal planes of (210), (002), (201), (211), (102), (220), (031), (112), (131), and (221) of the Fe_3_C phase (JCPDS 35-0772), respectively. The iron content of the Fe_3_C/N–C was analyzed through thermogravimetric analysis (TGA) (Figure S7). After aerobic pyrolysis of Fe_3_C/N–C, as expected, the residue was identified as Fe_2_O_3_ (Figure S8). As shown in Figure S4, based on the iron oxide mass after the TGA test (3.83 wt%), the iron content of the Fe_3_C/N–C was calculated as 2.8 wt%.

We further analyzed the sample through X-ray photoelectron spectroscopy (XPS) to understand the surface chemical composition and the electronic state of the elements in the Fe_3_C/N–C catalyst. From the survey spectrum of the Fe_3_C/N–C (Figure 2c), we can confirm the presence of C, N, O, and Fe elements, as expected [44]. The survey spectrum further reveals the presence of 3.65 atom% and 1.02 atom% of N and Fe, respectively, on the surface. The compound content calculated from XPS is lower than the actual amount, which can be attributed to the fact that XPS analysis has restricted access to the iron carbide nanoparticles and nitrogen atoms embedded inside the carbon layers due to the limited detecting depth in XPS (~5 nm) [45,46]. The high-resolution C 1s spectrum (Figure 2d) was fitted with four peaks corresponding to C–Fe (283.9 eV), C–C/C=C (284.6 eV), C–N (285.6), and C=O (288.0 eV) [47,48], suggesting the existence of graphite-like carbon (in an abundance of 50 atom%), nitrogen binding carbon (C–N), and iron binding carbon (Fe–C). Figure 2e shows the N 1s spectrum, which can be deconvoluted into four peaks, corresponding to the pyridinic N (398.8 eV), pyrrolic N (400 eV), graphitic N (401.1 eV), and oxidized-N (403.6), verifying the N-doping nature of the carbon structure [49,50]. It is worth mentioning that graphitic N sites (which, in our case, is around 45 atom%) particularly have the capability to improve the circulation of electrons due to their smaller diameter and higher electronegativity than carbon atoms [51]. Hence, graphitic carbon plus a significant amount of graphitic N in the network can combine together and encourage the breakage of the O-O bond in hydrogen peroxide [52]. As displayed in Figure 2f, the high-resolution Fe 2p XPS spectrum reveals two characteristic Fe 2p_3/2_ and Fe 2p_1/2_ peaks. The deconvoluted Fe 2p spectrum with peak positions at Fe^2+^ 2p_3/2_ (709.9 eV), Fe^3+^ 2p_3/2_ (712.5 eV), Fe^2+^ 2p_1/2_ (722.0 eV), and Fe^3+^ 2p_1/2_ (725.2 eV) demonstrates the successful formation of Fe_3_C over the porous carbon framework [53,54].

Generally speaking, surface area and porous structure play a crucial role in catalysis [55]. With the other conditions being the same, samples with a higher surface area show better catalytic performance [9,56]. The surface area and pore size distribution were studied through the nitrogen sorption isotherm (Figure S9). The isotherm exhibited an IUPAC-type IV shape. The specific surface area (S_BET_) and pore volume of Fe_3_C/N–C were 327 m^2^ g^−1^ and 0.17 cm^3^ g^−1^, respectively. Furthermore, Figure S10 displays the pore size distribution plot and proves the existence of dominant micropores and small mesopores [57]. The abundant microporous structure, if accessible, is vital to improving the catalytic activity because they can host the compact catalytic sites owing to the high surface-to-volume ratio [58,59,60]. Nevertheless, it is challenging for rich micropores in the membrane to exert their entire catalytic capacity due to the large diffusion resistance through pores below 2 nm in size. This challenge can be effectively addressed by the presence of mesopores, as shown in Figure S10, and macropores, as proven by the SEM image. Such a hierarchy in pores is valuable for catalysis, as micropores with rich active sites can be connected to meso- and/or macropores to balance the catalytic activity and mass transfer kinetics [36,61].

**Figure 3:**
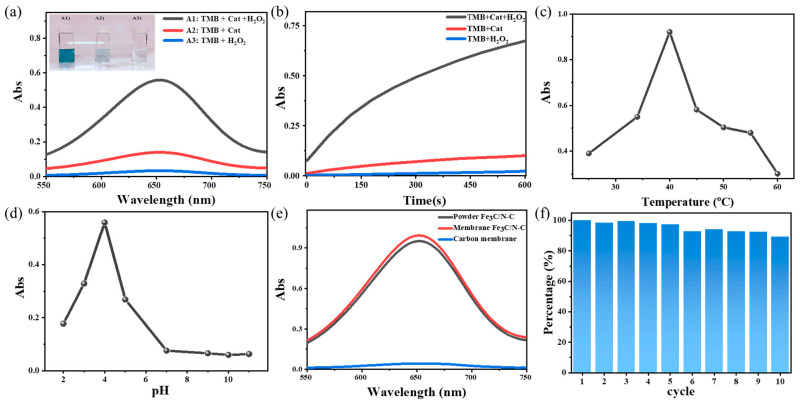
(**a**) UV-Vis absorbance and the corresponding optical photographs of the ox-TMB recorded in three systems (TMB + H_2_O_2_, TMB + Cat, TMB + H_2_O_2_ + Cat) in acetate buffer solutions at a pH value of 4.0. (**b**) Time-dependent absorbance spectra of ox-TMB at 652 nm in the three systems (TMB + H_2_O_2_, TMB + Cat, TMB + H_2_O_2_ + Cat). (**c**,**d**) Dependence of the peroxidase-like activity of the obtained Fe_3_C/N–C catalyst with varied temperature and pH values, respectively. (**e**) UV-Vis absorbance of the ox-TMB recorded with three catalysts (powder Fe_3_C/N–C, membrane Fe_3_C/N–C, pure carbon membrane) in acetate buffer solutions at a pH value of 4.0. (**f**) The stability test of the catalyst after 10 cycles of use.

**Mimetic peroxidase activity.** According to previous studies, carbon materials containing iron and nitrogen species illustrated enzyme-like catalytic properties [62,63]. For biosensors and other analytical applications, the optical properties of the sample were first monitored and investigated. The peroxidase-like activity of Fe_3_C/N–C was evaluated in the oxidation reaction of TMB in the presence of H_2_O_2_. In a typical process, the colorless liquid reaction mixture becomes blue with a characteristic absorbance peak emerging at 652 nm, originating from the oxidized TMB (termed ox-TMB), similar to what has been observed for the well-known horseradish peroxidase (HRP) [64,65]. The TMB solution, which only contained either H_2_O_2_ or the catalyst of Fe_3_C/N–C, did not show a distinguishable UV-Vis adsorbance at 652 nm, and the reaction system remained colorless and unchanged (curves and inset in Figure 3a). These results suggested that the oxidation reaction did not take place. Once the catalyst, H_2_O_2_, and TMB were all combined in the system, the blue color emerged (curve and inset A1 in Figure 3a). This proved that the Fe_3_C/N–C could decompose H_2_O_2_, which is responsible for triggering the oxidation reaction of TMB into ox-TMB that showed up as an absorption peak at 652 nm in the UV-Vis spectrum [66,67].

Several parameters, such as reaction time, pH, temperature, and the concentration of H_2_O_2_ and TMB, could affect the catalytic performance of Fe_3_C/N–C [11]. Figure 3b reveals the intensity of the absorbance peak at 652 nm of the mixtures of TMB + Cat (Fe_3_C/N–C), TMB + H_2_O_2_, and TMB + H_2_O_2_ + Cat systems after a reaction period of 10 min. The significant and continuous alteration in absorbance was detected in the TMB + H_2_O_2_ + Cat system as compared with TMB + Cat and TMB + H_2_O_2_, as the former needed a much longer time to reach a steady state than the latter two [68]. One of the crucial factors in a catalytic reaction is the temperature. In this study, the dependence of the catalytic activity of Fe_3_C/N–C on the reaction temperature was studied in the range from 25 to 60 °C. Figure 3c and Figure S11 demonstrate that the optimum reaction temperature for our peroxidase catalyst is 40 °C, which is close to the human body temperature, so it is more applicable for vitamin C detection in the biological samples, e.g., human serums. Regarding the drop in activity over 40 °C, similar phenomena have been illustrated by Biswas et al. [69]. Furthermore, the effect of pH on catalytic activity was investigated in the range of 2.0–11.0 (Figure 3d and Figure S12). In strong acidic media (at pH of 2.0), a pale blue color was obtained. At pH 3.0 and pH 5.0, the system revealed a slight blue color with 50% relative activity. A solid blue color was detected at pH 4.0, suggesting the best catalytic activity of the sample at pH ~4.0. According to previous studies, HRP also showed similar behavior [64]. Thus, the pH value for the further catalytic studies was chosen to be 4.0. The UV-Vis absorbance spectrum of TMB oxidation was assessed by changing the concentrations of TMB (0.02–1 mM) and H_2_O_2_ (7–80 mM). The tests showed that optimum absorbance occurred at 0.2 mM TMB (Figure S13) and 50 mM H_2_O_2_ (Figure S14). Furthermore, the performance of the catalyst in both membrane and powder states was compared with each other (Figure 3e), which illustrated that the catalytic activity of powder and membrane catalysts was practically the same. This observation proves that the membrane shape due of its low thickness did not retard the reaction kinetics, thanks to the hierarchical porous structure and high surface. In addition, the activity of the nitrogen-free pure carbon membrane, obtained from the carbonization of pure Balsa wood under the same condition, was measured as a control test; the result asserts that the presence of Fe_3_C nanoparticles and nitrogen doping is essential in this application. Due to the free-standing membrane shape of the Fe_3_C/N–C, it is easy to recycle it, thus reducing the cost by simple recycling. In Figure 3f, it is observed that the activity of our hierarchical porous membrane catalyst remains at 90% of the initial activity after 10 cycles, demonstrating its astonishing robustness in repeated use.

**Figure 4:**
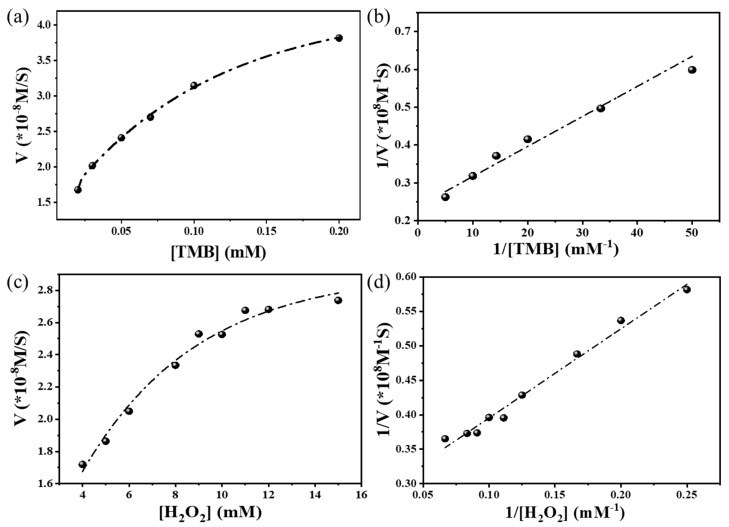
Steady-state kinetic experiments of Fe_3_C/N–C for catalytic tests. (**a**) The concentration of H_2_O_2_ was 50 mM and the TMB concentration varied. (**b**) Lineweaver–Burk plots for TMB substrate. (**c**) The concentration of TMB was 200 µM and the H_2_O_2_ concentration varied. (**d**) Lineweaver–Burk plots for H_2_O_2_ substrate. An amount of 20 μL of catalyst (3 mg·L^−1^) was used in this experiment conducted at room temperature.

The steady-state kinetic factors for the mimetic peroxidase reaction were determined by altering the concentration of one substrate while keeping the others constant. The catalytic performance of the Fe_3_C/N–C was profoundly probed through the kinetic analysis employing the TMB and H_2_O_2_ concentration as the variable. Figure 4a,c reveal the typical Michaelis–Menten curves; from the achieved curves, their Lineweaver–Burk plots were gained, as shown in Figure 4b,d. The initial velocity of ox-TMB was analyzed from the absorbance data, and the molar attenuation coefficient of TMB at 652 nm was 39,000 M^−1^cm^−1^ by using the Lambert–Beer (Equation (2)) law as shown below.

*A* = *εcb* (2)

where *A*, *ε*, *c*, and *b* are denoted as the absorbance, molar absorbance coefficient, substrate concentration, and the thickness of the sample, respectively. The Michaelis–Menten constant (*K_m_*) and maximum velocity (*V_max_*) were obtained from Equation (3).

(3) $$\frac{1}{V}=\frac{K_{m}}{V_{max}}.\frac{1}{\left[S\right]}+\frac{1}{V_{max}}$$

where *V* represents the initial reaction rate, *V_max_* represents the maximum initial rate, *K_m_* represents the Michaelis–Menten constant, and [*S*] represents the substrate concentration. It is known that the catalytic power of a sample depends on the *K_m_* and *V_max_* values [70,71]. The *K_m_* value indicates attraction among the enzyme and substrate, with a lower *K_m_* value representing a stronger affinity between the enzyme and substrate. The larger *V_max_* value proposes a better efficiency for TMB oxidation in the presence of hydrogen peroxide. In this study, the *K_m_* and *V_max_* values of the Fe_3_C/N–C catalyst for TMB were calculated to be 0.033 mM and 4.2 × 10^−8^ Ms^−1^, respectively. The *K_m_* value was about 15 times smaller than that of HRP (0.41 mM) [39], suggesting an improved affinity of the catalyst to TMB than that of HRP. This effect could stem from the high surface area, the hierarchical porous structure, and the presence of ultrafine Fe_3_C nanoparticles in Fe_3_C/N–C, which developed more active sites for TMB and a lower *K_m_* value [3]. The *K_m_* and *V_max_* values were compared with HRP and several other nanomaterials, as shown in Table S1. It is worth mentioning that less than 5 min was required to see the distinguishable color difference in the Fe_3_C/N–C system. These results suggest that Fe_3_C/N–C is efficient and needs a shorter time for colorimetric study than other similar mimetic peroxidases (usually more than 10 min). Such properties are vital factors for a rapid visual colorimetric test.

**Figure 5:**
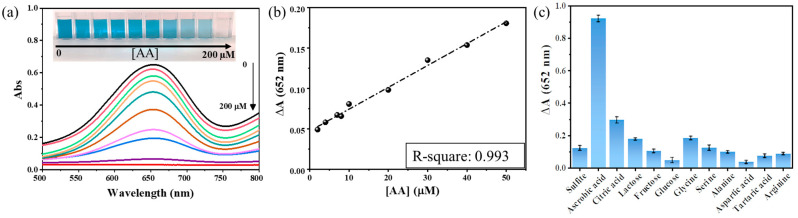
(**a**) Absorbance changes of the mixing solution consisting of TMB, the catalyst suspension, and H_2_O_2_ in the absence or presence of ascorbic acid and corresponding optical photographs. (**b**) Linear calibration plot to detect ascorbic acid. ΔA = A (652 nm, absence) − A (652 nm, ascorbic acid). (**c**) ΔA values of the Fe_3_C/N–C–TMB–H_2_O_2_ system at 652 nm in the presence of ascorbic acid or other interferential substances.

**Colorimetric detection of ascorbic acid using the peroxidase-like catalytic reaction of Fe_3_C/N–C.** Because of the intrinsic peroxidase property of the Fe_3_C/N–C, we designed a colorimetric system to detect AA in aqueous solutions, using the same substrate studied above, i.e., TMB and H_2_O_2_. The detection of vitamin C is according to its antioxidant nature of preventing oxidation via reducing the reactive free radicals [72,73]. Hence, the corresponding UV-Vis absorbance spectrum of the Fe_3_C/N–C–TMB–H_2_O_2_ system versus vitamin C concentration ([AA]) from 0 to 200 μM is presented in Figure 5a, and the linear relationship between ΔA (the difference between UV-Vis absorbance before and after adding AA) and [AA] was gained in the range of 2–50 μM (R^2^: 0.993) (Figure 5b). The limit of detection was around 2.64 µM, which is better than many other reports (Table S2).

To evaluate the selectivity of detection of AA by monitoring the absorbance of the reaction systems, vitamin C was replaced by various biologically relevant species such as sulfite, citric acid, glycine, serine, alanine, aspartic acid, tartaric acid, arginine, and saccharides like glucose, lactose, and fructose. The significant change in the absorbance intensity at 652 nm was not noticed after adding other interferential substrates. Figure 5c depicts the ΔA values of those systems. The considerable difference in the ΔA values among the mentioned interferences and vitamin C at similar concentrations can be clearly seen. These outcomes prove the outstanding specificity of our sensing process to the colorimetric detection of AA.

## 4. Conclusions

In summary, uniformly dispersed Fe_3_C nanoparticles were introduced into a hierarchically porous nitrogen-doped carbon membrane derived from a thin wood slice pre-coated by PIL and FeCl_3_ before carbonization. Owing to the unique interconnected and oriented porous structure, ultrafine nanoparticles, and nitrogen doping, the as-prepared Fe_3_C/N–C catalyst demonstrated outstanding intrinsic peroxidase-like catalytic activity with favorable stability and recyclability, which could be utilized to sensitively detect ascorbic acid over a broad concentration range with a low limit of detection (~2.64 µM). The detection system exhibited a high selectivity and anti-interference capacity to ascorbic acid. This study proposes a straightforward and effective way for preparing metal-containing heteroatom-doped porous carbon membranes, which can be generalized to synthesize various other functional carbonaceous materials such as artificial enzymes, revealing great potential in medicine, biosensing, and environmental fields.

## Data Availability

Data will be available upon request.

## Supplementary Materials

The following supporting information can be downloaded at https://www.mdpi.com/article/10.3390/nano13202786/s1. Additional results such as Figure S1: ^1^H-NMR of the PIL, Figure S2: UV−Vis absorbance spectra of the ox-TMB, Figure S3: Cross-sectional SEM images of Fe_3_C/N−C, Figure S4: Nanoparticle size distribution of Fe_3_C/N−C based on the TEM image, Figure S5: TEM image of Fe_3_C/N−C when PIL wasn’t used to disperse iron source, Figure S6: Particle size distribution of Fe_3_C/N−C when PIL wasn’t used to disperse iron source, Figure S7: TGA curve of Fe_3_C/N−C under air, Figure S8: PXRD diagram of the TGA residue of v, Figure S9: Nitrogen sorption isotherm of Fe_3_C/N−C, Figure S10: Pore size distribution plot of Fe_3_C/N−C, Figure S11: Temperature dependence plot of the peroxidase-like activity, Figure S12: pH dependence of the peroxidase-like activity, Figure S13: TMB concentration dependence of the peroxidase-like activity, Figure S14: H_2_O_2_ concentration dependence of the peroxidase-like activity, Table S1: Comparison of the apparent Michaelis constant (Km) and maximum reaction rate (Vmax) between our work and other groups’ work, Table S2: Analytical characteristics of different colorimetric AA measuring system. References [74,75,76,77,78,79,80,81,82] are cited in the supplementary materials.
