# Supplementary material for: Facile Fabrication of Wood-Derived Porous Fe3C/Nitrogen-Doped Carbon Membrane for Colorimetric Sensing of Ascorbic Acid
Source: Nanomaterials (Basel). 2023 Oct 18;13(20):2786. doi: 10.3390/nano13202786 (PMC10609461; doi:10.3390/nano13202786)
Supplement: Supplementary file 1 [file nanomaterials-13-02786-s001.zip › nanomaterials-2658861-supplementary.pdf]

Supplementary Information

Facile fabrication of wood-derived porous Fe_3C/nitrogen-doped carbon membrane for colorimetric sensing of ascorbic acid

*Sadaf Saeedi Garakani 1, Miao Zhang 1, Dongjiu Xie 2, Anirban Sikdar 1, Kanglei Pang 1 and Jiayin Yuan 1,**

1 Department of Materials and Environmental Chemistry, Stockholm University, 10691 Stockholm, Sweden

2 Department for Electrochemical Energy Storage, Helmholtz-Zentrum Berlin für Materialien und Energie, Hahn-Meitner Platz 1, 14109 Berlin, Germany

** Correspondence: jiayin.yuan@mmk.su.se*

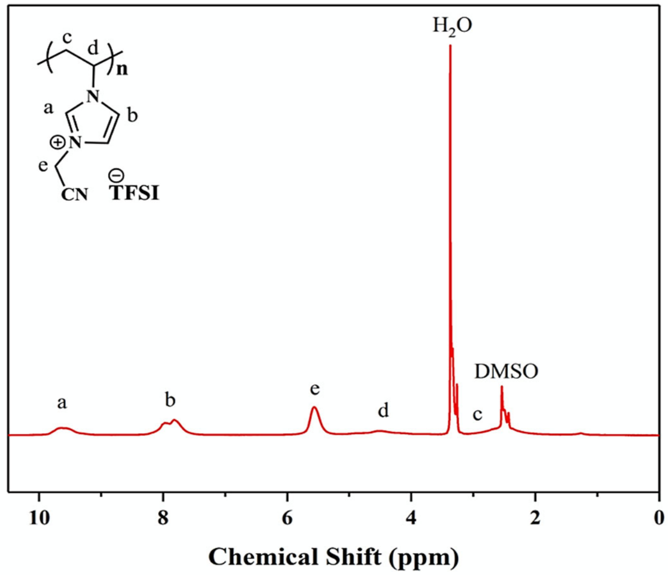

Figure S1 ^{1}H-NMR of the PIL poly(1-cyanomethyl-3-vinylimidazoluim bis(trifluoromethane sulfonyl)imide) (PCMVImTFSI), which was used for the porous membrane fabrication. NMR solvent: DMSO-d_6.

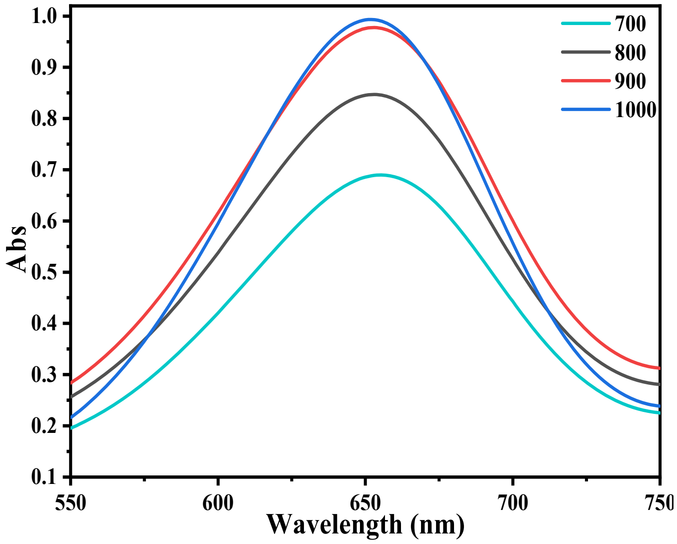

Figure S2 UV−Vis absorbance spectra of the ox-TMB recorded in three systems (TMB + H_2O_2 + Cat) prepared at different carbonization temperature, in acetate buffer solution at pH of 4.0.

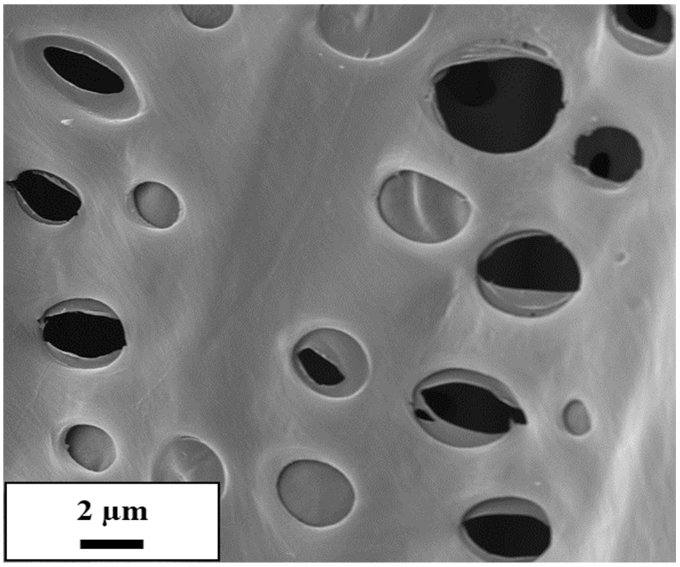

Figure S3 Cross-sectional SEM images of Fe_3C/N−C.

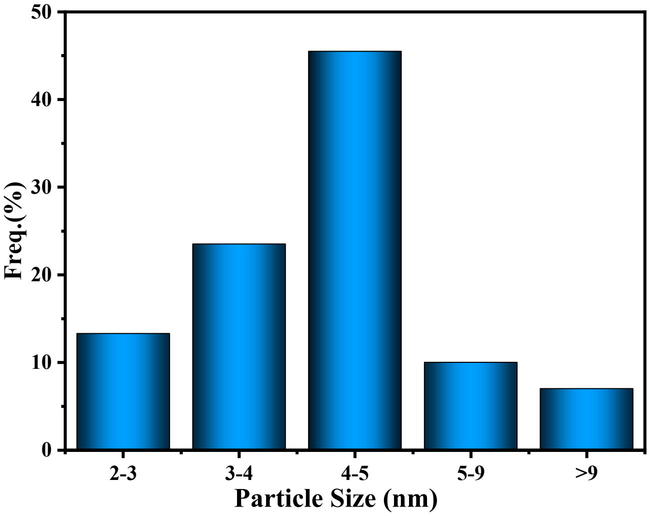

Figure S4 Nanoparticle size distribution of Fe_3C/N−C based on the TEM image in Figure 1.

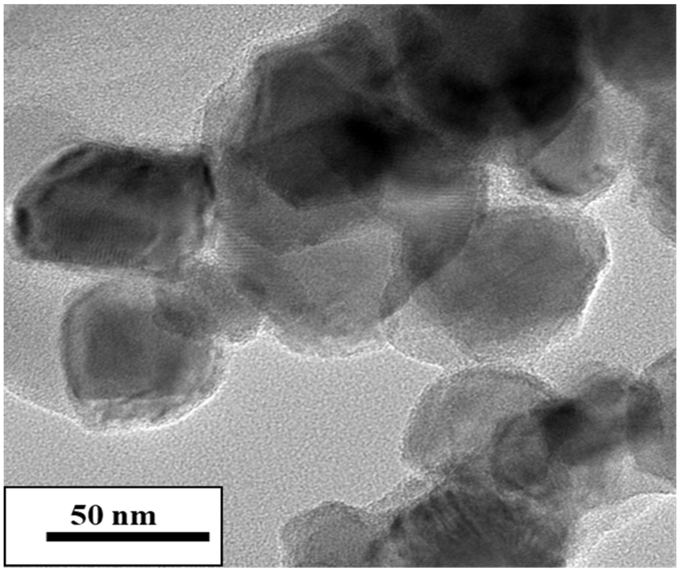

Figure S5 TEM image of Fe_3C-C when PIL wasn't used to disperse iron source and the agglomeration of big iron carbide particles was observed.

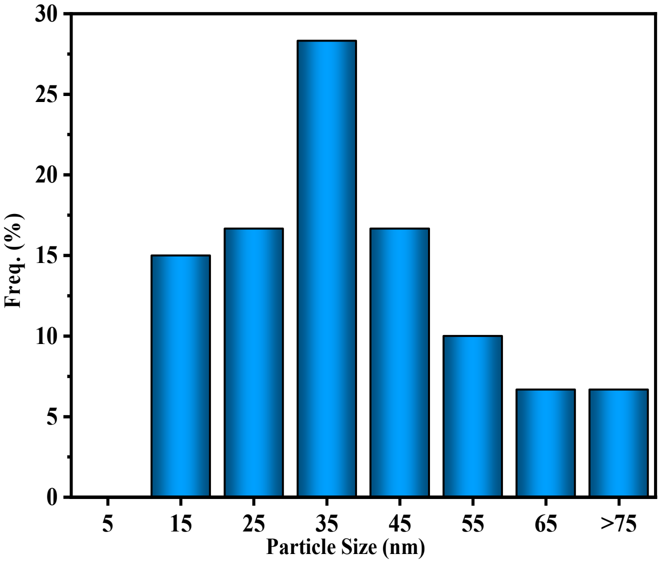

Figure S6 Particle size distribution of Fe_3C-C when PIL wasn't used to disperse iron source.

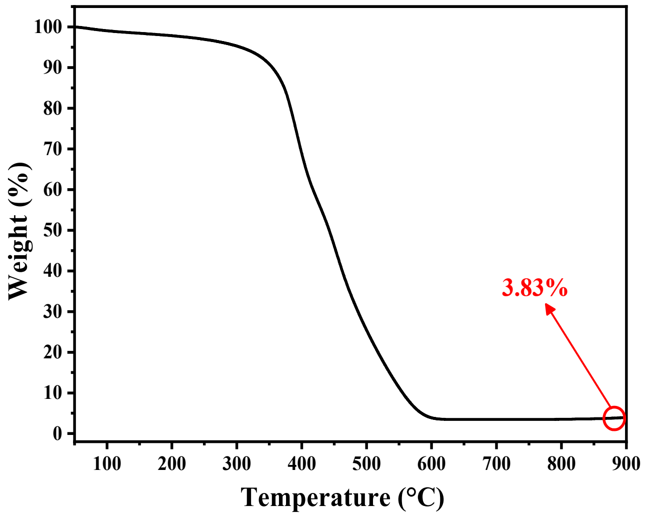

Figure S7 TGA curve of Fe_3C/N−C under air.

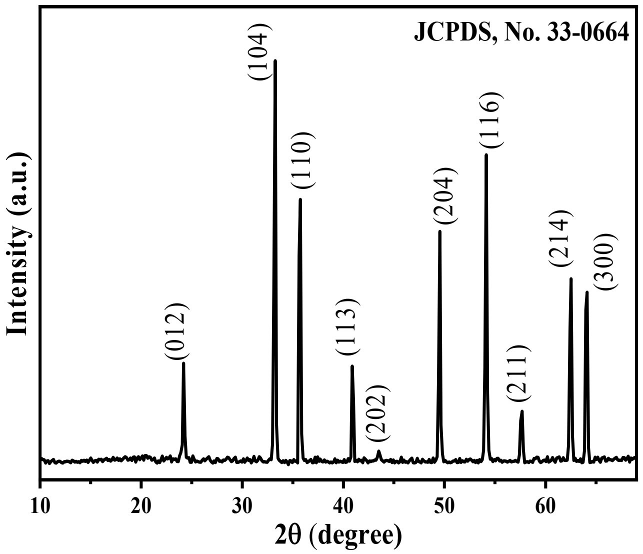

Figure S8 PXRD diagram of the TGA residue of Fe_3C/N−C after heating to 900 ºC in air, indicative of an α-Fe_2O_3 phase.

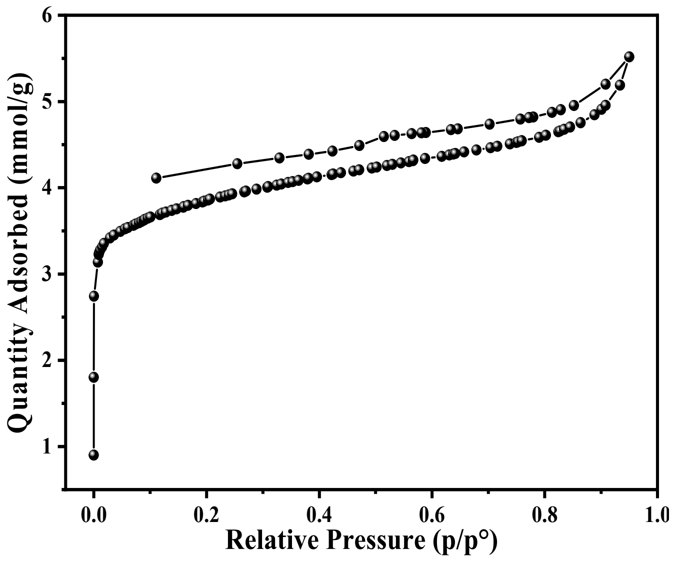

Figure S9 Nitrogen sorption isotherm of Fe_3C/N−C measured at 77 K.

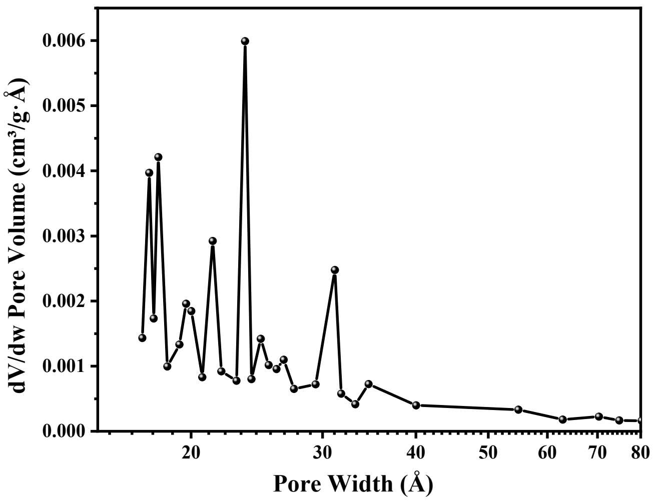

Figure S10 Pore size distribution plot of Fe_3C/N−C.

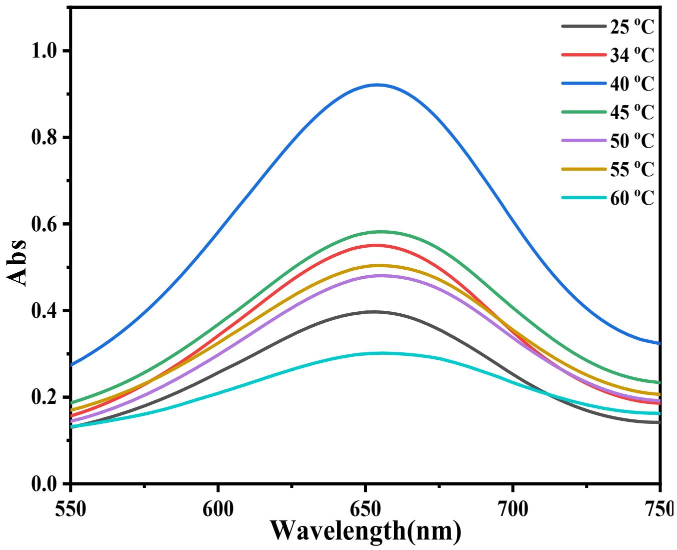

Figure S11 Temperature dependence plot of the peroxidase-like activity of the obtained Fe_3C/N−C catalyst.

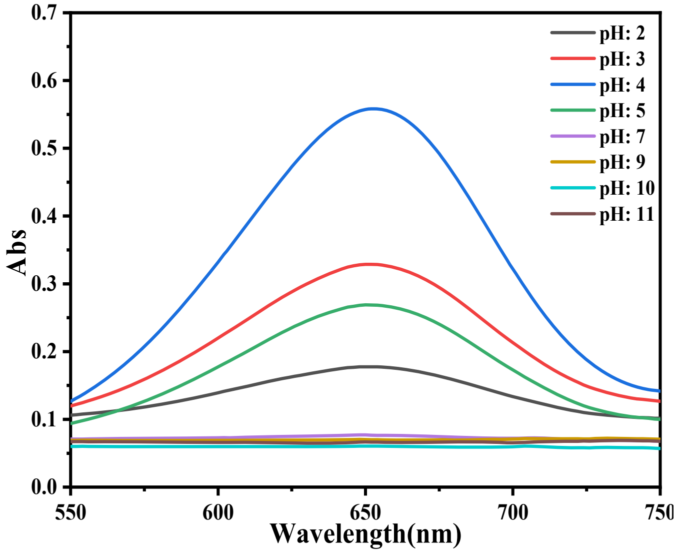

Figure S12 Dependence of the peroxidase-like activity of the obtained Fe_3C/N−C catalyst on pH.

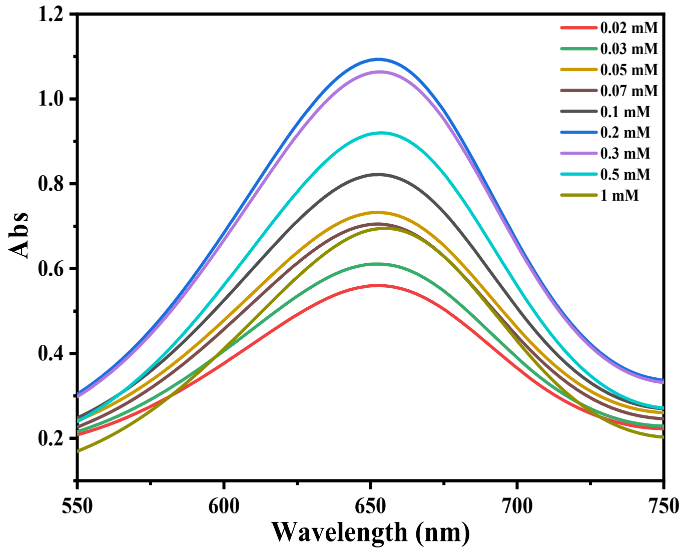

Figure S13 Dependence of the peroxidase-like activity of the obtained Fe_3C/N−C catalyst on TMB concentration.

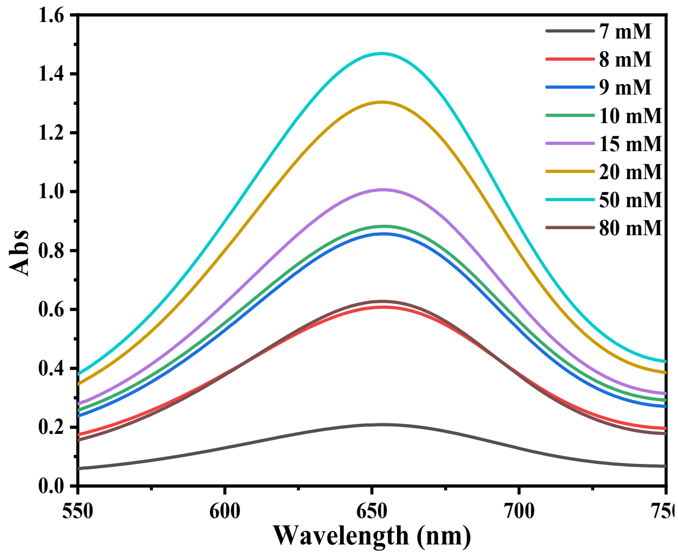

Figure S14 Dependence of the peroxidase-like activity of the obtained Fe_3C/N−C catalyst on H_2O_2 concentration

Table S1 Comparison of the apparent Michaelis constant (K_m) and maximum reaction rate (V_{max}) between our work and other groups' work.

Catalyst	Substrate	K_m (mM)	V_{max} (10^{-8} M s^{-1})	Reference
Fe_3O_4	TMB<br>H_2O_2	0.098<br>154	3.44<br>9.78	[1]
Au/Co_3O_4-CeO_x NCs	TMB<br>H_2O_2	0.1219<br>0.2724	0.8577<br>0.3898	[2]
WSe_2 nanosheets	TMB<br>H_2O_2	0.433<br>19.53	1.43<br>2.22	[3]
Fe_3C/NGr	TMB<br>H_2O_2	0.25<br>38.42	8.26<br>13.06	[4]
AKCN	TMB<br>H_2O_2	0.60<br>0.79	6.78<br>2.53	[5]
HRP	TMB<br>H_2O_2	0.41<br>0.74	1.12<br>1.11	[4]
**Fe_3C/N-C**	**TMB**<br>**H_2O_2**	**0.033**<br>**4.9**	**4.2**<br>**3.84**	**This work**

Table S2 Analytical characteristics of different colorimetric AA measuring system.

Catalyst	LOD (μM)	Range	Reference
MOF-808	15	30-1030 μM	[6]
MIL-68	6	30-485 μM	[7]
MIL-53(Fe)	15	28.6-190.5 μM	[8]
CNT	20	80-136 μM	[9]
SQE	10	50-425 μM	[10]
Silver nanoparticles (AgNPs)	82.8	1-4 mM	[11]
MoO_3	90	1-100 mM	[12]
**Fe_3C/N-C**	**2.64**	**2-50 μM**	**This work**

References

1. Gao, L.; Zhuang, J.; Nie, L.; Zhang, J.; Zhang, Y.; Gu, N.; Wang, T.; Feng, J.; Yang, D.; Perrett, S.; et al. Intrinsic Peroxidase-like Activity of Ferromagnetic Nanoparticles. *Nat. Nanotechnol.* **2007**, *2*, 577–583, doi:10.1038/nnano.2007.260.

2. Liu, H.; Ding, Y.; Yang, B.; Liu, Z.; Liu, Q.; Zhang, X. Colorimetric and Ultrasensitive Detection of H2O2 Based on Au/Co3O4-CeOx Nanocomposites with Enhanced Peroxidase-like Performance. *Sensors Actuators, B Chem.* **2018**, *271*, 336–345, doi:10.1016/j.snb.2018.05.108.

3. Chen, T.M.; Wu, X.J.; Wang, J.X.; Yang, G.W. WSe2 Few Layers with Enzyme Mimic Activity for High-Sensitive and High-Selective Visual Detection of Glucose. *Nanoscale* **2017**, *9*, 11806–11813, doi:10.1039/c7nr03179c.

4. Wu, S.; Huang, H.; Feng, X.; Du, C.; Song, W. Facile Visual Colorimetric Sensor Based on Iron Carbide Nanoparticles Encapsulated in Porous Nitrogen-Rich Graphene. *Talanta* **2017**, *167*, 385–391, doi:10.1016/j.talanta.2017.02.003.

5. Zhang, P.; Sun, D.; Cho, A.; Weon, S.; Lee, S.; Lee, J.; Han, J.W.; Kim, D.P.; Choi, W. Modified Carbon Nitride Nanozyme as Bifunctional Glucose Oxidase-Peroxidase for Metal-Free Bioinspired Cascade Photocatalysis. *Nat. Commun.* **2019**, *10*, 1–14, doi:10.1038/s41467-019-08731-y.

6. Zheng, H.Q.; Liu, C.Y.; Zeng, X.Y.; Chen, J.; Lü, J.; Lin, R.G.; Cao, R.; Lin, Z.J.; Su, J.W. MOF-808: A Metal-Organic Framework with Intrinsic Peroxidase-Like Catalytic Activity at Neutral PH for Colorimetric Biosensing. *Inorg. Chem.* **2018**, *57*, 9096–9104, doi:10.1021/acs.inorgchem.8b01097.

7. Zhang, J.W.; Zhang, H.T.; Du, Z.Y.; Wang, X.; Yu, S.H.; Jiang, H.L. Water-Stable Metal–Organic Frameworks with Intrinsic Peroxidase-like Catalytic Activity as a Colorimetric Biosensing Platform. *Chem. Commun.* **2014**, *50*, 1092–1094, doi:10.1039/c3cc48398c.

8. Ai, L.; Li, L.; Zhang, C.; Fu, J.; Jiang, J. MIL-53(Fe): A Metal-Organic Framework with Intrinsic Peroxidase-like Catalytic Activity for Colorimetric Biosensing. *Chem. - A Eur. J.* **2013**, *19*, 15105–15108, doi:10.1002/chem.201303051.

9. Wang, Z.; Liu, J.; Liang, Q.; Wang, Y.; Luo, G. Carbon Nanotube-Modified Electrodes for the Simultaneous Determination of Dopamine and Ascorbic Acid. *Analyst* **2002**, *127*, 653–658, doi:10.1039/b201060g.

10. Alizadeh, N.; Ghasemi, F.; Salimi, A.; Hallaj, R.; Fathi, F.; Soleimani, F. Polymer Nanocomposite Film for Dual Colorimetric and Fluorescent Ascorbic Acid Detection Integrated Single-Cell Bioimaging with Droplet Microfluidic Platform. *Dye. Pigment.* **2020**, *173*, 107875, doi:10.1016/j.dyepig.2019.107875.

11. Ferreira, D.C.M.; Giordano, G.F.; Soares, C.C.D.S.P.; De Oliveira, J.F.A.; Mendes, R.K.; Piazzetta, M.H.; Gobbi, A.L.; Cardoso, M.B. Optical Paper-Based Sensor for Ascorbic Acid Quantification Using Silver Nanoparticles. *Talanta* **2015**, *141*, 188–194, doi:10.1016/j.talanta.2015.03.067.

12. Li, R.; An, H.; Huang, W.; He, Y. Molybdenum Oxide Nanosheets Meet Ascorbic Acid: Tunable Surface Plasmon Resonance and Visual Colorimetric Detection at Room Temperature. *Sensors Actuators, B Chem.* **2018**, *259*, 59–63, doi:10.1016/j.snb.2017.12.058.
